# Medicaid Managed Long-Term Services and Supports in relation to Nursing Home Staffing and Care Diversion

**DOI:** 10.1093/geroni/igaf122.3810

**Published:** 2025-12-31

**Authors:** Alison Trinkoff, Jennifer Choi, Michael Lepore

**Affiliations:** University of Maryland School of Nusing, Baltimore, Maryland, United States; Sejong University, Sejong University, Seoul-t’ukpyolsi, Republic of Korea; University of Massachusetts Amherst, Amherst, Massachusetts, United States

## Abstract

**Background:**

In 2020, Medicaid accounted for approximately 61% of total U.S. spending on long-term services and supports (LTSS), or $200 billion. As the primary LTSS payer, Medicaid faces significant financial pressures from rising demand. In response, US states have implemented Medicaid-Managed LTSS programs, shifting from fee-for-service models to managed care systems, yet few studies have evaluated the impact of these shifts on care.

**Purpose:**

This examined two outcomes: care diversion from institutions, and changes in nursing home staffing levels that could mediate the programs’ impact on care quality.

**Methods:**

The study used data from six states that implemented MLTSS between 2010-2014. We examined the impact on care diversion and staffing levels using a staggered difference-in-differences framework that captures dynamic effects over time.

**Results:**

The proportion of Medicaid residents in nursing homes significantly decreased following MLTSS program implementation. Also, the proportion of residents with other payer sources increased, along with a significant rise in total nursing hours per resident day. Cumulative gains across nurse staffing categories contributed to the significant rise in overall staffing, supporting program goals of improving care quality. For diversion, we identified a shift in nursing home resident composition by payer groups, without significant changes in the total number of residents, potentially reflecting diversion of Medicaid beneficiaries to alternative care settings. This suggests that the observed changes reflect a reallocation in resident mix rather than fluctuations in overall nursing home censuses.

**Conclusions:**

Findings offer new evidence on the potential beneficial effects of MLTSS implementation across multiple US states.

